# Microbial DNA methylation links rumen transcriptional activity to methane emission divergence in dairy cows

**DOI:** 10.1002/imt2.70153

**Published:** 2026-07-14

**Authors:** Xinyue Zhang, Guirong Xu, Liwei Zhao, Ye Tao, Jiawei Zhang, Cameron E. F. Clark, Yangyi Hao, Zhijun Cao, Shengli Li, Wei Wang

**Affiliations:** ^1^ State Key Laboratory of Animal Nutrition and Feeding, College of Animal Science and Technology China Agricultural University Beijing China; ^2^ Shanghai Biozeron Biotechnology Co. Ltd. Shanghai China; ^3^ Gulbali Institute Charles Sturt University North‐Wagga Wagga Australia

## Abstract

Genome‐resolved methylomics uncovers the first DNA methylation atlas of the dairy cow rumen microbiome. Microbial DNA methylation profiles diverge between low‐ and high‐ methane‐emitting dairy cows despite limited taxonomic differences. Differential methylation is linked to transcriptional variation in genes involved in carbon metabolism, hydrogen utilization, and methanogenesis.

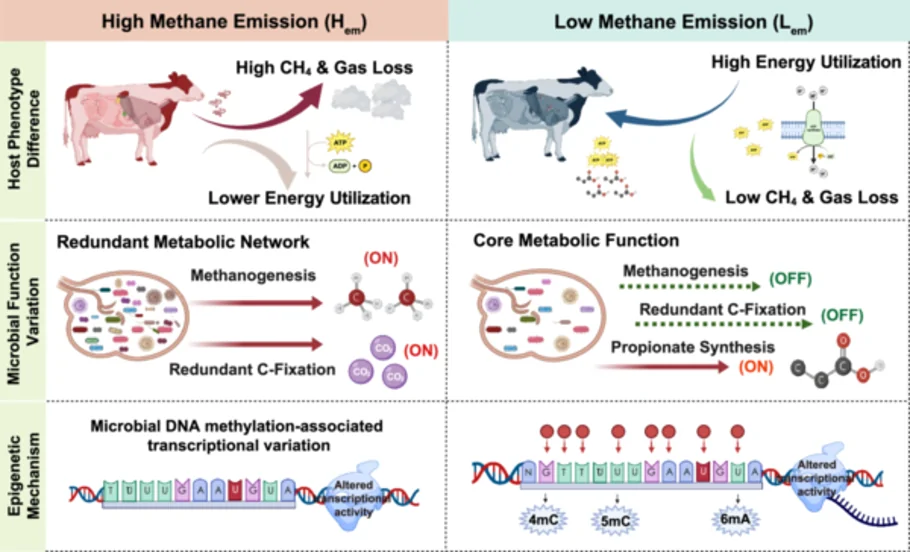

## AUTHOR CONTRIBUTIONS

**Xinyue Zhang**: Conceptualization; methodology; data curation; software; formal analysis; visualization; writing—original draft; writing—review and editing. **Guirong Xu**: Investigation; data curation; writing—review and editing. **Liwei Zhao**: Software; visualization; writing—review and editing. **Ye Tao**: Methodology; software; validation; writing—review and editing. **Jiawei Zhang**: Software; validation; writing—review and editing. **Cameron E. F. Clark**: Writing—review and editing. **Yangyi Hao**: Writing—review and editing. **Zhijun Cao**: Writing—review and editing. **Shengli Li**: Writing—review and editing. **Wei Wang**: Conceptualization; methodology; investigation; writing—original draft; writing—review and editing; project administration; funding acquisition; supervision; resources.

## CONFLICT OF INTEREST STATEMENT

Ye Tao is the CEO of Shanghai Biozeron Biotechnology Co. Ltd. The other authors declare no conflicts of interest.

## ETHICS STATEMENT

All animal procedures were conducted in accordance with national guidelines for animal care and were approved by the Animal Ethics Committee of China Agricultural University, Beijing, China (Permission ID: AW90506202‐1‐01).

To the Editor,

Methane (CH_4_) produced during ruminal fermentation is a major source of greenhouse gas emissions from dairy production and represents an energetic loss to the host animal [1, 2]. Considerable inter‐individual variation in methane emission exists among dairy cows, even under comparable feeding and management conditions. Understanding the biological basis of this variation is important for developing targeted methane‐mitigation strategies [3, 4].

Previous studies have linked enteric methane emission to rumen microbial composition and hydrogen metabolism [5, 6]. However, differences in microbial taxonomic abundance inconsistently explain variation in methane output across animals [7, 8], highlighting a potential role for microbial functional regulation beyond community structure alone. Recent advances in prokaryotic epigenetics provide a new conceptual framework for understanding this microbial functional regulation. DNA methylation is one of the most widespread epigenetic modifications in prokaryotes and can influence transcriptional activity, genome defense, and metabolic adaptation without altering DNA sequence [9, 10]. Whether microbial DNA methylation contributes to rumen methanogenesis in complex ecosystems remains largely unknown.

Here, we integrated Oxford Nanopore Technologies (ONT) long‐read sequencing [11] to systematically characterize the methylome of the dairy cow rumen microbiome at the metagenome‐assembled genome (MAG) level. By linking microbial DNA methylation patterns to transcriptional and metabolic differences, we aim to advance the current understanding from a traditional species abundance‐based theory to an epigenetic association‐based framework. Furthermore, we establish the first genome‐wide DNA methylation atlas of the rumen microbiome from dairy cows, providing a mechanistic foundation for epigenetics‐informed methane mitigation and precision microbiome engineering in low‐carbon livestock production.

## METABOLIC REALLOCATION CHARACTERIZES LOW METHANE‐EMITTING DAIRY COWS

**Figure 1:**
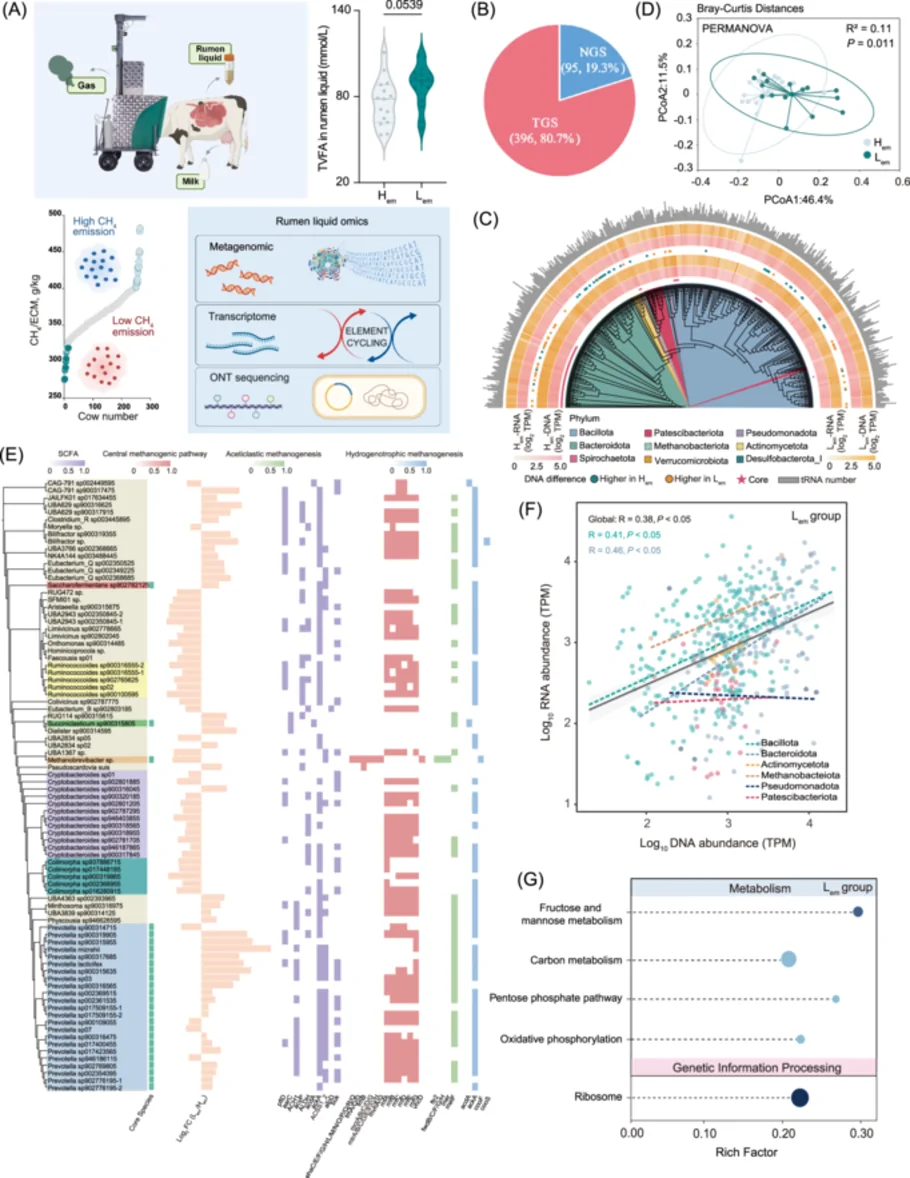
Functional difference of the rumen microbiome associated with divergent methane emission phenotypes. (A) Experimental design and rumen metabolic phenotype. High (H_em_) and low (L_em_) methane‐emitting individuals (*n* = 15 each) were selected from 264 Holstein cows using the GreenFeed system. Rumen liquid was subjected to metagenomic, metatranscriptomic, and ONT‐based microbial DNA methylation sequencing. Violin plot shows total volatile fatty acid (TVFA) concentrations in rumen fluid (H_em_ group vs. L_em_ group, *p* = 0.0539). (B) Numbers of nonredundant species‐level metagenome‐assembled genomes (MAGs) reconstructed from third‐generation sequencing (TGS) and next‐generation sequencing (NGS) data. (C) Phylogenetic tree of 491 species‐level MAGs based on GTDB taxonomy, with annotations for phylum classification, DNA abundance differences (H_em_ group vs. L_em_ group), tRNA gene copy number, and core genome classification. (D) Principal coordinate analysis (PCoA) based on Bray‐Curtis distances of rumen microbial community composition (PERMANOVA, *R* ^2^ = 0.11, *p* = 0.011, permutations = 999). (E) Genome functional profiling of core microbial taxa, displaying contributions to short‐chain fatty acid (SCFA) metabolism and key methanogenesis pathways, including central, acetoclastic, and hydrogenotrophic routes. (F) Correlations between metagenomic abundance (DNA) and metatranscriptomic expression (RNA) across MAGs in L_em_ group. (G) KEGG pathway enrichment analysis containing > 100 DEGs in the Lem group (*p* < 0.01).

To investigate the microbial mechanisms underlying methane emission divergence, enteric methane production from 264 Holstein dairy cows was continuously monitored using the GreenFeed system [12]. Based on individual methane production intensity (CH_4_/energy corrected milk, g/kg ECM), cows were stratified into high‐emission (H_em_ group; *n* = 15; milk yield: 45.26 ± 1.6) and low‐emission (L_em_ group; *n* = 15; milk yield: 46.73 ± 2.7) groups. Rumen fluid samples were subjected to volatile fatty acid (VFA) quantification and multi‐omics analyses, including metagenomics, metatranscriptomics, and epigenetic profiling (Figure 1A).

Compared with the H_em_ group, the L_em_ group exhibited significantly reduced CH_4_, CO_2_, and H_2_ emission (Supporting Information S1: Figure S1A–C and Supporting Information S1: Table S1), accompanied by a trend toward higher total volatile fatty acid (TVFA) concentration, particularly propionate and valerate (Figure 1A, Supporting Information S1: Figure S1D, Supporting Information S1: Table S2). Correlation analysis further revealed significant negative correlations between methane emission and ruminal VFA profiles (Supporting Information S1: Figure S1E), indicating a competitive allocation of metabolic flux between methanogenesis and VFA synthesis pathways [13]. However, the overall taxonomic composition exhibited minimal differences at the domain level (Supporting Information S1: Figure S1F,G). While metagenomic alpha diversity did not differ significantly between groups, metatranscriptomic diversity was markedly lower in the L_em_ group (Supporting Information S1: Figure S1H, Shannon index, *p* < 0.05). Together, these results suggest that methane emission divergence could not be fully explained by microbial taxonomic composition alone, but rather by differences in microbial functional involvement.

## A GENOME‐RESOLVED REFERENCE CATALOG REVEALS HIGH‐QUALITY RECONSTRUCTION OF THE RUMEN MICROBIOME

To overcome read length and genome assembly limitations of traditional next‐generation sequencing (NGS), we constructed a high‐quality reference genome catalog by integrating third‐generation sequencing (TGS) and NGS data. After filtering low‐quality MAGs, 491 species‐level (including 18 novel species) and 817 strain‐level (including 34 novel strains) nonredundant MAGs were obtained based on average nucleotide identity (ANI) thresholds of 95% and 99%, respectively. Among the 491 species‐level MAGs, 396 were assembled from TGS data and 95 were from NGS data (Figure 1B). Notably, TGS‐assembled genomes exhibited improved completeness, lower contamination, more 16S rRNA gene copies, fewer contigs, and higher N50 values (Supporting Information S1: Figure S2A–D), providing a high‐quality genomic reference framework for accurate metatranscriptomic mapping and high‐resolution identification of DNA methylation sites.

Phylogenetic analysis based on 491 species‐level MAGs revealed that *Bacillota* and *Bacteroidota* dominated the rumen microbiome (Figure 1C). Beta diversity analysis suggested the separation between H_em_ and L_em_ groups (Figure 1D). Functional profiling based on KEGG Orthology (KO) composition showed that species within the same phylum shared similar functional potentials (Supporting Information S1: Figure S2E). The L_em_ group exhibited a more streamlined functional architecture and higher network stability (Supporting Information S1: Figure S2F). Furthermore, by referencing the rumen core microbial catalog [14], core taxa in this study were predominantly assigned to the genus *Prevotella* (Figure 1E). However, no significant differences were observed between the two groups in the abundance of key genes involved in VFA biosynthesis, central methanogenesis, acetoclastic methanogenesis, and hydrogenotrophic methanogenesis pathways, suggesting that gene abundance alone could not explain methane emission differences and implicating higher‐order regulatory mechanisms.

## METATRANSCRIPTOMICS REVEALS FUNCTIONAL DIFFERENCES IN THE RUMEN MICROBIOME

To achieve a more precise characterization of rumen microbial gene functions, we mapped metatranscriptomic reads to the MAG‐based gene catalog constructed in this study. This genome‐resolved quantification approach effectively eliminates interference from environmental DNA and species with incomplete genome assemblies, thereby more accurately reflecting the transcriptional activity of core microbial functional genes. Correlation analysis across 491 MAGs showed that microbial abundance was not consistently positively correlated with transcriptional activity (Figure 1F and Supporting Information S1: Figure S3A), highlighting limitations of abundance‐based interpretations.

Among 351,326 expressed genes detected in this study, differential expression analysis identified 8298 upregulated and 20,398 downregulated genes in the L_em_ group (Figure S3B). Pathway enrichment analysis (differentially expressed genes [DEGs] >100 and enrichment significance *p* < 0.01) revealed that multiple metabolic pathways were significantly enriched in the H_em_ group, including carbon fixation and methane metabolism (Supporting Information S1: Figure S3C,D), suggesting a more functionally redundant metabolic network. In contrast, the L_em_ group exhibited fewer enriched pathways (Figure 1G and Supporting Information S1: Figure S3E) and a more specialized transcriptional profile in core metabolic functions [15]. Further functional attribution at the genus level revealed distinct roles of core and top 10 non‐core taxa (Supporting Information S1: Figure S3F–G). Although *Prevotella* was a dominant contributor to the functional potential of the microbiome in both groups, it was more involved in the biosynthesis of secondary metabolites and biosynthesis of antibiotics in the H_em_ group, whereas it was more engaged in fundamental processes such as ribosome biogenesis in the L_em_ group. Furthermore, *Methanobrevibacter* in the H_em_ group was enriched in methane metabolism, whereas *Succiniclasticum* and *Pseudoscardovia* in the L_em_ group primarily contributed to ribosome biogenesis.

Carbohydrate‐active enzymes (CAZymes) were more abundant in the H_em_ group (Supporting Information S1: Figure S4A). Hydrogenase profiling revealed that [NiFe]‐hydrogenases were predominantly associated with *Methanobrevibacter*, whereas [FeFe]‐hydrogenases were enriched in *Colimorpha*, *Moryella*, *Porcincola*, and *Succiniclasticum* (Supporting Information S1: Figure S4B). Differential abundance analysis further revealed that the [FeFe]‐hydrogenase‐carrying *Succiniclasticum sp900315805* was significantly enriched in the L_em_ group, whereas the [NiFe]‐hydrogenase‐carrying *Methanobrevibacter tgs.All.semibin2.987* was predominantly involved in the H_em_ group (Supporting Information S1: Figure S4C), uncovering a redistribution of carbon and hydrogen metabolic fluxes. Finally, we delineated an interactive metabolic network linking methanogenesis with acetate, propionate, and butyrate biosynthesis (Supporting Information S1: Figure S4D). By integrating shared metabolic intermediates, this metabolic network clearly illustrated the distribution and contribution of key genes across fermentation and methanogenesis pathways across different microbial phyla.

## MICROBIAL DNA METHYLATION LANDSCAPE REVEALS EPIGENETIC DIVERGENCE

**Figure 2:**
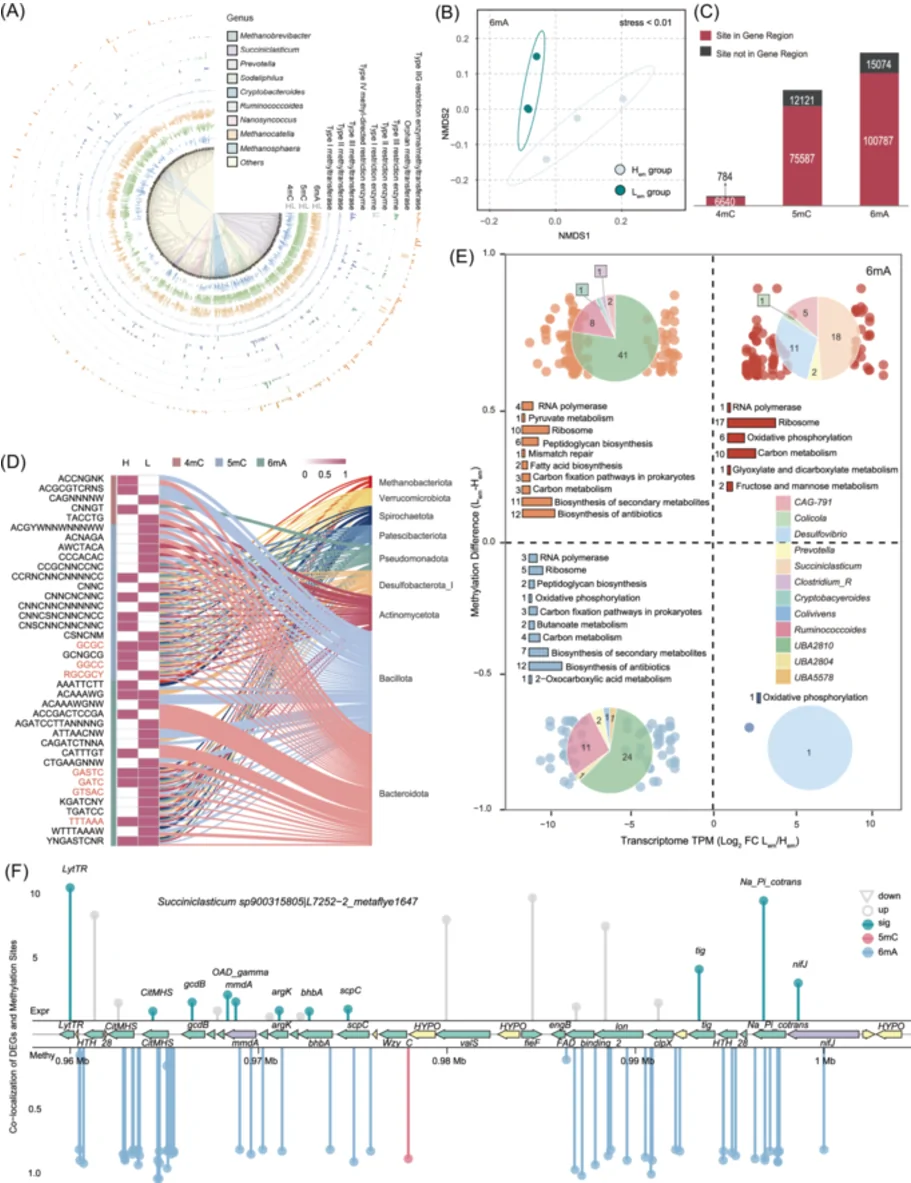
Microbial DNA methylation landscape and its associations with transcriptional activity in the rumen microbiome. (A) Phylogenetic tree of 491 species‐level metagenome‐assembled genomes (MAGs), annotated with the distribution of microbial DNA methylation motifs (4mC, 5mC, 6mA) and methyltransferase‐restriction‐modification (R‐M) system types across dominant rumen microbial genera. (B) Nonmetric multidimensional scaling (NMDS) analysis of genome‐wide methylation profiles in microbial DNA methylation of 6mA. (C) Genomic distribution of microbial DNA methylation sites across genic and intergenic regions. (D) Sankey diagram showing taxonomic assignment of microbial DNA methylation motifs and their distribution across rumen microbial taxa. (E) Quadrant‐based integration of transcriptomic and methylomic data for 6 mA. The *x*‐axis represents transcriptional changes (Log_2_ FC L_em_ group/H_em_ group), and the *y*‐axis represents microbial DNA methylation differences (L_em_ group–H_em_ group > 0.6). Each dot represents a differentially expressed gene (DEG), colored by the corresponding microbial genus. Pie charts show the taxonomic composition of DEGs in each quadrant, and bar plots display the enriched KEGG pathways for genes correlated with transcriptional and microbial DNA methylation changes. (F) Genomic co‐localization analysis of microbial DNA methylation sites and DEGs in *Succiniclasticum sp900315805*.

Here, the first comprehensive genome‐wide DNA methylation landscape of the rumen microbiome in dairy cows was constructed and linked to enteric CH_4_ phenotype (Figure 2A), providing an epigenetic framework to explain enhanced energy utilization in low methane‐emitting individuals. Based on the genome‐wide methylation profiles of three major prokaryotic DNA methylation types (4mC, 5mC, and 6mA), non‐metric multidimensional scaling (NMDS) analysis based on Bray–Curtis distances revealed the separation between H_em_ and L_em_ groups (Figure 2B and Supporting Information S1: Figure S5A,B, Stress <0.05), indicating distinct microbial DNA methylation patterns associated with methane emission phenotype. Subsequently, de novo motif identification, followed by annotation against the REBASE database [16], identified 43 nonredundant microbial DNA methylation motifs. Among these, only 7 (16.3%) matched known motifs, whereas 36 (83.7%) were novel (Supporting Information S1: Figure S5C). The novel motifs were distributed across 1161 microbial species, approximately 26.61% of which represented previously undescribed taxa. Notably, even among MAGs carrying known motifs, nearly 28.05% were assigned to previously uncharacterized species. These findings indicate that current reference databases capture only a limited fraction of the methylation diversity present in the rumen microbiome and suggest that uncultivated and poorly characterized microbial species may represent an important reservoir of previously unrecognized methylation systems. Although the biological functions of these motifs remain unknown, DNA methylation in prokaryotes has been linked to restriction‐modification (R‐M) systems, genome defense, DNA replication, and transcriptional regulation. Consequently, these newly identified motifs may represent previously unrecognized mechanisms involved in microbial adaptation and ecological interactions within the rumen ecosystem. Further characterization of the methyltransferases responsible for these modifications will be required to investigate their functional significance.

Genomic distribution analysis showed that microbial DNA methylation sites and motifs exhibited distinct patterns between genic and intergenic regions (Figure 2C and Supporting Information S1: Figure S5D). Across gene bodies, 4mC, 5mC, and 6mA modifications were distributed across the entire transcriptional unit (0%–100%) without clear positional bias across microbial phyla (Supporting Information S1: Figure S5E), suggesting their potential involvement in genome‐wide functional processes. Taxonomic assignment demonstrated that several conserved motifs, including GCGC, ACAAAWG, GASTC, GATC, TTTAAA, and YNGASTCNR, were shared across both groups, whereas others displayed strong species specificity and were associated with distinct microbial taxa (Figure 2D). These findings suggest that DNA methylation constitutes a previously underappreciated layer of functional diversification within the rumen microbiome.

## MICROBIAL DNA METHYLATION IS ASSOCIATED WITH TRANSCRIPTIONAL REGULATION

Integrative analysis of methylome and metatranscriptome data on the MAG level using a quadrant‐based framework revealed coordinated shifts in microbial DNA methylation and transcription. Among DEGs, 0.12% (4mC), 11.37% (5mC), and 21.65% (6mA) exhibited co‐localization between DEGs and microbial DNA methylation, indicating 5mC and 6mA as the predominant methylation types associated with differential gene expression in the rumen microbiome. Functional attribution of differentially methylated genes showed that under 5mC involvement, genes downregulated in the L_em_ group were primarily derived from *Prevotella* and *Bifidobacterium*, enriched in carbon metabolism, carbon fixation, and biosynthetic pathways, whereas genes upregulated in the L_em_ group were associated with *Desulfovibrio* and *RUG420*, enriched in ribosome biogenesis and carbon metabolism pathways (Supporting Information S1: Figure S5F). Among genes associated with 6mA methylation, genes increased in the H_em_ group were mainly assigned to *Ruminococcoides* and *UBA2810*, enriched in secondary metabolite and antibiotics biosynthesis, while genes decreased in H_em_ group were linked to *Desulfovibrio* and *Succiniclasticum*, enriched in ribosome biogenesis and carbon metabolism (Figure 2E). Together, these results indicate that DNA methylation may systematically contribute to microbial transcriptional activity for functional differentiation of the rumen microbiome. To delineate the functional role of the detected microbial DNA methylation, we classified and quantified methyltransferases (MTases), including type I–IV, IIG R‐M, and orphan MTases [9] across the 491 MAGs (Supporting Information S1: Figure S5G). Type I–III MTases were numerically dominant, reflecting the prevalence of R‐M systems in the rumen microbiome. Notably, the concurrent presence of orphan MTases indicates methylation independent of restriction activity, supporting a dual role in host defense and gene regulation.

## GENE‐LEVEL EPIGENETIC VARIATION MAY CONTRIBUTE TO METABOLIC FLUX

To further define methylation‐associated variation at the single‐genome level, we performed genomic co‐localization analysis between DEGs and microbial DNA methylation sites. A representative circular genome of *Pseudoscardovia radai* (2.2 Mbp) shows comprehensive genomic features (Supporting Information S1: Figure S5H), including GC content, coding sequence distribution (CDS), transcriptional variation (RNA‐FC), microbial DNA methylation motifs (CCGCNNCCNC, RGCGCY, GCGC, CSNCNM, CNNCNNCNNNNNC), and key functional genes involved in carbohydrate degradation (*GH101*), sugar metabolism and propionate production (*pflB, fadD3*), methylation regulation (*rumA*), and energy synthesis (*atpA*).

Co‐localization analysis revealed a pronounced clustering of microbial DNA methylation signals with functional genes. Specifically, dense 5mC and 6mA modifications were frequently observed near genes involved in carbohydrate transport (*sus*) and amino acid/energy metabolism (*gcv*) in *Prevotella lacticifex* (Supporting Information S1: Figure S6A) and *Prevotella sp900316565* (Supporting Information S1: Figure S6B). Notably, the associations between microbial DNA methylation and gene expression followed either species‐ or site‐specific patterns rather than uniformly activating or repressing. For example, increased 6mA methylation significantly enhanced transcription of the key propionate synthesis gene *mmdA* in *Succiniclasticum sp900315805* (Figure 2F), promoting propionate production and reducing hydrogen availability for methanogenesis. In contrast, elevated 6mA methylation markedly suppressed expression of the hydrogen metabolism gene *nifJ* in *Ruminococcoides sp902773155* (Supporting Information S1: Figure S6C), thereby reducing free hydrogen supply and inhibiting methanogenic activity. Furthermore, the energy metabolism gene *accC* in *Desulfovibrio sp900319575* was positively associated with both 5mC and 6mA modifications (Supporting Information S1: Figure S6D), showing cooperative epigenetic involvement of metabolic pathways.

Together, differences in microbial composition and the abundance of key methanogenesis‐related genes were relatively limited between methane‐emission phenotypes, whereas methylation profiles differed and were associated with changes in gene expression. These observations indicate that microbial DNA methylation may provide additional insights into transcriptional regulation and metabolic differences within the rumen microbiome that are not readily captured by community composition alone, ultimately influencing host methane emission and energy utilization efficiency.

Several limitations should be considered when interpreting these findings. Although DNA methylation may contribute to transcriptional difference, the current data do not allow the direction of causality to be established. Differences in methylation patterns may also arise as a consequence of shifts in microbial physiological state, growth dynamics, ecological interactions, or metabolic activity associated with divergent methane‐emission phenotypes. In addition, the functional interpretation of methylation sites in complex microbial communities remains challenging because promoters and other functional genomic elements are difficult to accurately predict across diverse and largely uncultivated microbial genomes represented by MAGs. Consequently, the present study focused on genome‐wide associations between methylation and transcriptional activity rather than assigning regulatory functions to specific genomic elements. Future studies employing targeted characterization of methyltransferases, functional manipulation of methylation‐associated genes, and in vitro validation experiments will be required to explore whether the observed methylation patterns directly influence gene expression and methane‐related metabolic processes.

## Supporting information


**Figure S1:** Comparison of gas emissions, rumen fermentation profiles, and microbial diversity between high (H_em_) and low (L_em_) methane‐emitting dairy cows.
**Figure S2:** Quality assessment of metagenome‐assembled genomes (MAGs) and comparative functional analysis of the rumen microbiome.
**Figure S3:** Metatranscriptomic functional difference and metabolic flux distribution in the rumen microbiome of dairy cows with divergent methane emissions.
**Figure S4:** Carbohydrate‐active enzymes and hydrogenase difference in the rumen microbiome of dairy cows with divergent methane emissions.
**Figure S5:** Microbial DNA methylation motif discovery and gene‐level epigenetic regulation in the rumen microbiome.
**Figure S6:** The co‐localization of differentially expressed genes and microbial DNA methylation in the rumen microbiome with divergent methane emissions.

**Table S1:** Milk yield and gas emission in H_em_ and L_em_ groups.
**Table S2:** Volatile fatty acid concentrations in H_em_ and L_em_ groups.

## Data Availability

The data that supports the findings of this study are available in the supplementary material of this article. The metagenomic, metatranscriptomic, and Oxford Nanopore Technologies sequencing data produced in this study are available at the NCBI Sequence Read Archive with the BioProject: PRJNA1466632 (https://www.ncbi.nlm.nih.gov/bioproject/PRJNA1466632/). MAG dataset and methylation profiles are available at: 10.6084/m9.figshare.32610396. The code supporting the findings of this study is available at: https://github.com/zd200572/RMM. Supplementary materials (methods, tables, figures, graphical abstract, slides, videos, Chinese translated version and update materials) may be found in the online DOI or iMeta Science http://www.imeta.science/.
