# Supplementary material for: Microbial DNA methylation links rumen transcriptional activity to methane emission divergence in dairy cows
Source: Imeta. 2026 Jul 14;5(4):e70153. doi: 10.1002/imt2.70153 (PMC13519090; doi:10.1002/imt2.70153)
Supplement: Supplementary file 1 [file IMT2-5-e70153-s002.docx]

Supporting Information to

**Microbial DNA methylation links rumen transcriptional activity to methane emission divergence in dairy cows**

**Running title**: microbial methylation and methane

Xinyue Zhang^1^, Guirong Xu^1^, Liwei Zhao^1^, Ye Tao^2^, Jiawei Zhang^1^, Cameron EF Clark^3^, Yangyi Hao^1^, Zhijun Cao^1^, Shengli Li^1^, Wei Wang^1^*

^1^State Key Laboratory of Animal Nutrition and Feeding, College of Animal Science and Technology, China Agricultural University, Beijing 100193, China

^2^Shanghai Biozeron Biotechnology Co., Ltd, Shanghai 201800, China

^3^Gulbali Institute, Charles Sturt University, North-Wagga Wagga, NSW 2650, Australia

^*^Correspondence: [wei.wang@cau.edu.cn](mailto:wei.wang@cau.edu.cn) (Wei Wang)

**METHODS**

**Experimental design and sample collection**

This study was conducted at a commercial dairy farm in Dongying, China (37.95° N, 118.87° E), housing 5,805 lactating Holstein dairy cows. The trial lasted from October 11, 2024 to January 16, 2025. Animals were fed a total mixed ration (TMR) three times daily at 07:00, 13:00, and 19:00 h, with *ad libitum* access to fresh water. During the experimental period, enteric methane (CH_4_) emissions were continuously monitored using the GreenFeed system (C-lock Inc., Rapid City, South Dakota, USA), and milk yields were recorded daily using the DeLaval Rotary E500 milking system (Tetra Laval Group, Tumba, Sweden). At the end of the monitoring period, 264 cows with complete methane-recording data were ranked according to their average daily CH_4_ emission intensity (g/kg energy corrected milk). Finally, a total of 30 cows, including the 15 highest and 15 lowest CH_4_ emission intensity individuals (DIM: 194.53 vs. 197.60; Parity: 2.00 vs. 1.87), were selected for subsequent rumen fluid and milk sampling.

Enteric gas emissions, including CH_4_, carbon dioxide (CO_2_), oxygen (O_2_), and hydrogen (H_2_), were quantified using the GreenFeed (C-lock Inc., Rapid City, South Dakota, USA) [1]. Briefly, GreenFeed units dispensed alfalfa pellets as bait (30 g per 40 s, maximum 8 drops per visit) to attract cows for gas emission measurement. Each cow was fitted with a radio-frequency identification (RFID) ear tag to record the timing and duration of each visit. Visits lasting longer than 3 min were considered valid records. To reduce temporal auto-correlation among repeated measurements, a minimum interval of 5 h was required between valid visits from the same cow, with a maximum of four measurements per cow per day. During the experimental period, at least 20 valid visits per cow were obtained. Individual methane emissions were calculated as the arithmetic mean of all valid GreenFeed observations obtained from each cow. To assess the stability and reliability of methane phenotyping and to evaluate the accuracy of the H_EM_ and L_EM_ classifications, a sensitivity analysis was performed using individual methane phenotypes estimated from mixed-effects model-derived BLUP.

Cows were milked three times daily at 06:00, 12:00, and 18:00 h using a DeLaval Rotary E500 milking system (Tetra Laval Group, Tumba, Sweden). Milk samples from each milking session were collected and mixed at a ratio of 4:3:3, then the milk composition was detected using a CombiFossTM7 analyzer (Foss Analytical A/S, Hilleroed, Denmark). Energy corrected milk (ECM) was calculated using the following formula:

$$\text{ECM=0.327×milk yield+12.95×milk fat yield+7.2×milk protein yield}$$

Rumen fluid samples were collected 2 h after morning feeding using an oral intubation (Wuhan Anscitech Animal Husbandry Technology Co., Ltd., Wuhan, China). To minimize saliva contamination, the first 50 mL of rumen liquid was discarded, and the subsequent 15 mL was immediately frozen in liquid nitrogen and stored at -80°C for further analysis.

**Measurement of rumen fermentation parameters**

Volatile fatty acid (VFA) concentrations, including acetate, propionate, butyrate, valerate, isobutyrate, and isovalerate, were measured according to the method described by Kong et al [2]. Briefly, rumen fluid samples were thawed at room temperature and centrifuged at 16,000 × g for 15 min at 4°C (5804R centrifuge, Eppendorf, Oldenburg, Germany). Subsequently, 1.5 mL supernatant was transferred into a new tube containing 300 μL of 25% (v/v) metaphosphoric acid (Beijing Yili Fine Chemicals, Beijing, China). The mixture was incubated on ice for 30 min and centrifuged again at 16,000 × g for 10 min at 4°C. The clarified supernatant was transferred into gas chromatography vials, and VFA concentrations were detected using an Agilent G6890N gas chromatograph (Agilent Technologies, Santa Clara, CA, USA). Individual VFAs were identified and quantified according to retention times and peak areas relative to external standards.

**DNA extraction and sequencing**

Total genomic DNA was extracted from rumen fluid samples using Qiagen DNeasy PowerSoil Pro Kit (Qiagen, Hilden, Germany) according to the manufacturer’s instructions. For samples yielding less than 500 ng DNA per extraction, repeated extractions were performed and pooled to obtain at least 5 μg DNA for downstream analyses. DNA concentration and purity were evaluated using a NanoDrop One spectrophotometer (Thermo Fisher Scientific, Waltham, MA, USA) and a Qubit dsDNA HS Kit (Thermo Fisher Scientific, Waltham, MA, USA). High-quality DNA samples (OD260/280 = 1.8-2.2, OD260/230 ≥ 2.0) were used for library construction.

Libraries for next-generation sequencing (NGS) were prepared using the MGIEasy Genomic DNA Library Preparation Kit (MGI Tech, Shenzhen, China), quantified using the Qubit dsDNA HS Assay Kit on a Qubit Fluorometer (Thermo Fisher Scientific, Waltham, MA, USA), and sequenced on the DNBSEQ-T7 platform (Shanghai Biozeron Biotech Co., Ltd.) in paired-end 150-bp mode. For third-generation sequencing (TGS), genomic DNA libraries were prepared using the SQK-NBD114-96 Ligation Sequencing Kit (Oxford Nanopore Technologies, Oxford, UK), loaded onto FLO-PRO114M Nanopore flow cells (R10.4.1), and sequenced on the PromethION 2 Solo platform.

**RNA extraction and metatranscriptome sequencing**

Total RNA was extracted from rumen fluid samples using the E.Z.N.A.^®^ Soil RNA Mini Kit (Omega Bio-tek, Norcross, GA, USA) according to the manufacturer’s instructions. Residual genomic DNA was removed using DNase I (TaKaRa, Otsu, Shiga, Japan). RNA integrity and quality were assessed using an Agilent 2100 Bioanalyser (Agilent Technologies, Santa Clara, CA, USA), and RNA concentration was measured using a NanoDrop ND-2000 spectrophotometer (NanoDrop Technologies, Wilmington, DE, USA). High-quality RNA samples (OD260/280 = 1.8-2.2, OD260/230≥2.0, RIN≥6.5, 28S:18S≥1.0, >10 μg) were used for library construction [3].

Metatranscriptome libraries were constructed using 5 μg total RNA following standard RNA library preparation procedures. Ribosomal RNA was removed using the Ribo-Zero^TM^ rRNA Removal Kits (Illumina, San Diego, CA, USA), and the remaining RNA was fragmented using fragmentation buffer. Subsequently, cDNA synthesis, end repair, A-tailing, and adapter ligation were performed according to the manufacturer’s instructions. Libraries containing inserts of approximately 200-300 bp were size selected using 2% Low Range Ultra Agarose gels and amplified using Phusion DNA polymerase for 15 PCR cycles. The final libraries were sequenced on the DNBSEQ-T7 platform with PE150 mode.

**Sequence quality control**

Raw NGS data were quality-filtered using Trimmomatic (<http://www.usadellab.org/cms/uploads/supplementary/Trimmomatic>) to remove adaptor sequences and low-quality reads [4]. Reads passing quality control were subsequently mapped against the Bos taurus reference genome (NCBI) using BWA-MEM algorithm (v0.7.17, <http://bio-bwa.sourceforge.net/bwa.shtml>) with parameters “-M -k 21 -t 16” to remove host-derived sequences. The remaining high-quality non-host reads were retained as clean reads for downstream analyses. Raw TGS data were filtered using fastplong (v0.22) with the parameters “ -q 5 -l 300” to obtain high-quality reads for subsequent analyses [5].

**Metagenomic assembly, MAG reconstruction and abundance calculation**

Clean short-read metagenomic sequences (NGS) were assembled *de novo* into contigs using MEGAHIT with the parameter “--min-contig-len 500” [6]. Contig abundance profiles were calculated using “jgi_summarize_bam_contig_depths” script implemented in MetaBAT2 (v2.17, https://bitbucket.org/berkeleylab/metabat) [7] based on BAM alignment files generated using BWA-MEM and SAMtools (v1.22.1, <http://www.htslib.org/>). Clean long-read sequences (TGS) were assembled using MetaFlye (v2.9.5, https://github.com/fenderglass/Flye). MAGs were reconstructed using MetaBAT2, Semibin2 (v2.2.0, https://github.com/BigDataBiology/SemiBin) and VAMB (v5.0.2, https://github.com/RasmussenLab/vamb) with default parameters (minimum contig length ≥ 1500 bp).

For quality assessment, MAGs were evaluated using CheckM2 (v1.0.2) with the lineage_wf workflow. MAGs with completeness ≥ 50% and contamination ≤ 10% were retained for downstream analyses. Redundant MAGs were dereplicated using dRep (v3.4.2) at 95% average nucleotide identity (ANI), and representative MAGs with the highest quality scores (completeness - 5 × contamination) were retained [8]. Taxonomic classification of MAGs was performed using GTDB-Tk (v2.3.2, <https://gtdb.ecogenomic.org/>), and MAGs lacking taxonomic assignments were excluded from subsequent analyses. The abundance of MAGs was estimated based on metagenomic read mapping using CoverM (v0.7, https://github.com/wwood/CoverM). Mapped reads were normalized by both MAG length and total sequencing depth to obtain length- and depth-normalized relative abundance values. These normalized values were used for downstream statistical analysis and visualization. A phylogenetic tree of MAGs was constructed using FastTree implemented in PhyloPhlAn 3 and visualized using the Interactive Tree of Life (iTOL) platform.

**Functional annotation of MAGs**

Open reading frames (ORFs) in MAGs were predicted using Prodigal (v2.6.3) [9]. Functional annotation of ORFs was performed against the Kyoto Encyclopedia of Genes and Genomes (KEGG) database using KofamScan (https://www.genome.jp/tools/kofamkoala/) and the HMMSEARCH program in the HMMER package. Carbohydrate-active enzymes (CAZymes) were annotated using the dbCAN3 [10] pipeline (https://pro.unl.edu/dbCAN2/) with HMMER and DIAMOND [11] tools under default parameters. Hydrogenase genes were identified by searching ORFs against the HydDB [12] database using DIAMOND with the parameters “--evalue 1e-5 --id 70”. To identify restriction enzymes and DNA methyltransferases (MTases), all ORFs were first searched against the Conserved Domain Database (CDD, v3.20) in NCBI using RPS-BLAST (v2.12.0 and e-value < 1×e^-5^). MTases were further identified using InterProScan (v5.76-107.0), and genes containing the domains cd21179, COG0350, KOG3191, pfam05869, pfam12047, PRK10904, PRK11524, TIGR00589, TIGR00675, TIGR01712 and TIGR02987 were extracted and annotated against the REBASE database (https://rebase.neb.com/rebase/rebase.html, e-value < 1×e^-5^) using the DIAMOND “blastp” module.

**Diversity and differential abundance analyses**

Alpha diversity indices were calculated using Mothur (v.1.48.1-1) [13]. Beta diversity was evaluated using Bray-Curtis distance matrices based on the relative abundance of MAGs and visualized by principal coordinates analysis (PCoA), while differences in microbial community structure were assessed by permutational multivariate analysis of variance (PERMANOVA) [14] using the vegan package (v2.6-4) in R. Differential abundance analysis for MAGs and visualization were performed using STAMP (v2.1.3, <https://beikolab.cs.dal.ca/software/STAMP>).

Transcript abundance was quantified using Salmon (v1.10.1). Gene abundance profiles were normalized as TPM, correcting for gene length and sequencing depth (alignment length ≥ 50 bp and sequence identity > 95%) [15]. The TPM is calculated as follows:

$$TPM=\frac{N_{g}/L_{g}}{\sum_{j} {(N}_{j}/L_{j})}\times{10}^{6}$$

where Ng is the read count, i.e., number of reads mapped to gene g; and Lg is the gene length, i.e., the number of nucleotides in the g gene. The index j stands for the set of all genes involved in a catalog, and g is an index indicating a particular gene.

Differentially expressed genes (DEGs) between the L_em_ and H_em_ groups were identified using edgeR (v3.24.3) in R [16] based on the criteria of |log_2_ (fold change)| > 2 and the false discovery rate (FDR) < 0.05. Raw *P*-values were corrected by the Benjamini-Hochberg algorithm to obtain FDR value. Genes with accumulated TPM abundance across six samples < 1 were discarded in further analysis. Functional enrichment analysis of DEGs was performed using KOBAS (<http://kobas.cbi.pku.edu.cn/home.do>) against the KEGG database. Pathways with Bonferroni-corrected *P* values < 0.05 were considered significantly enriched.

**Metagenomic DNA methylation analysis**

Raw Pod5 files generated from the PromethION 2 Solo platform were basecalled using Dorado with the super-accuracy model (dna_r10.4.1_e8.2_400bps_sup@v4.3.0; kit SQK-NBD114-96) on NVIDIA RTX A4000 GPUs. Basecalled reads were demultiplexed using Dorado (v1.3.1, https://software-docs.nanoporetech.com/dorado/latest/). Reads were aligned to the dereplicated MAGs using minimap2 [17]. DNA methylation calling was subsequently performed using MicrobeMod (v1.1.0) with the “call_methylation” module and the parameters “-m 6mA,5mC,4mC -methylation_confidence_threshold 0.66 -percent_methylation_cutoff 0.66 --min_strand_coverage 5” [18]. Once methylated sites were called from “call_methylation” module, bedtools (v2.30.0, <https://github.com/arq5x/bedtools)> software package was introduced to map the methylated sites to genes of MAGs. Differential methylation analysis was conducted at both base-level and gene-level resolution. Conserved methylation motifs were identified using MEME Suite with motif widths ranging from 4 to 14 bp [19]. Motif that does not exist in the REBASE database (http://rebase.neb.com/) was defined as novel motif. Beta diversity analysis of multi-type methylation profiles was performed using Bray-Curtis distance matrices followed by non-metric multidimensional scaling (NMDS) analysis in the vegan (v2.6-4) package of R. Stress values were used to evaluate ordination fit, and values < 0.05 were considered indicative of an excellent fit.

**Statistical analysis**

Phenotypic data, including gas emissions and rumen fermentation parameters, were analyzed using Welch’s t-test in GraphPad Prism (v9.0, GraphPad Software, San Diego, CA, USA). Data normality was assessed using the Shapiro-Wilk test implemented in R (v4.3.0). Because most variables significantly deviated from a normal distribution (*P* < 0.05), Spearman's rank correlation analysis was used to evaluate associations among microbial abundance and gene expression profiles using R software. For analyses involving multiple microbial taxa or genes, raw P values were adjusted for multiple comparisons using the Benjamini-Hochberg false discovery rate (FDR) procedure, and correlations with FDR-adjusted *P* values < 0.05 were considered statistically significant.

**SUPPLEMENTARY FIGURES**

**FIGURE S1. Comparison of gas emissions, rumen fermentation profiles, and microbial diversity between high (H_em_) and low (L_em_) methane-emitting dairy cows.** (A-C) Comparison of gas emission intensity between H_em_ and L_em_ groups, including CH_4_ (A), CO_2_ (B), and H_2_ (C) production. (D) Concentrations of individual volatile fatty acids (VFAs) in rumen fluid between the H_em_ and L_em_ groups. (E) Correlation analysis between methane emission and ruminal VFA profiles. (F-G) Microbial taxonomic composition at the domain level based on metagenomic (MG) data (F) and metatranscriptomic (MT) data (G). (H) Comparative analysis of MG and MT alpha diversity (Shannon index) between the H_em_ and L_em_ groups.

**FIGURE S2. Quality assessment of metagenome-assembled genomes (MAGs) and comparative functional analysis of the rumen microbiome.** (A-D) Comparison of genome assembly quality between MAGs recovered from third-generation sequencing (TGS) and next-generation sequencing (NGS), including completeness and contamination (A), 16S rRNA gene copy number (B), contig number (C) and N50 values (D). (E) Principal coordinate analysis (PCoA) based on KEGG Orthology (KO) composition, colored by the dominant rumen microbial phylum. (F) Functional interaction networks of the rumen microbiome in the high methane-emitting (H_em_) and low methane-emitting (L_em_) groups based on metagenomic (MG) data.

**FIGURE S3. Metatranscriptomic functional difference and metabolic flux distribution in the rumen microbiome of dairy cows with divergent methane emissions.** (A) Correlations between metagenomic abundance (DNA) and metatranscriptomic expression (RNA) across MAGs in H_em_ group. (B) Volcano plot of differentially expressed genes between H_em_ and L_em_ groups. Dashed horizontal and vertical lines indicate the thresholds of FDR = 0.05 and |log_2_FC| = 2, respectively. (C) KEGG pathway enrichment analysis of differentially expressed genes (differentially expressed genes > 100, enrichment *P* < 0.01) in H_em_ group. (D-E) Differential pathway enrichment analysis based on metatranscriptomic data (differentially expressed genes > 100, *P* < 0.01) in the high methane‑emitting (H_em_) group (D) and low-methane-emitting (L_em_) group (E). Box size represents gene counts, and color intensity indicates enrichment significance. (F-G) Number of differentially expressed genes and average Log_2_ FC (L_em_/H_em_) of core and top 10 non‑core genera in the H_em_ group (F) and L_em_ group (G).

**FIGURE S4.** **Carbohydrate-active enzymes and hydrogenase difference in the rumen microbiome of dairy cows with divergent methane emissions.** (A) Comparative abundance of carbohydrate-active enzymes (CAZymes) between the H_em_ and L_em_ groups. (B) Species distribution and relative abundance of hydrogenase types. (C) Differential abundance of hydrogenase-carrying species between the H_em_ and L_em_ groups. (D) Interactive metabolic network linking rumen methanogenesis with acetate, propionate, and butyrate biosynthesis pathways.

**FIGURE S5. Microbial DNA methylation motif discovery and gene-level epigenetic regulation in the rumen microbiome.** (A-B) Non-metric multidimensional scaling (NMDS) analysis of genome-wide methylation profiles in microbial DNA methylation of 4mC (A), and 5mC (B), respectively. (C) Proportions of known and novel non-redundant microbial DNA methylation motifs. (D) Genomic distribution of motifs across genic and intergenic regions. (E) Distribution of 4mC, 5mC, and 6mA modifications across gene bodies (0-100% of transcriptional units). (F) Quadrant-based integration of transcriptomic and methylomic data for 5mC. The x-axis represents transcriptional changes (Log_2_ FC L_em_ group/H_em_ group), and the y-axis represents microbial DNA methylation differences (L_em_ group - H_em_ group > 0.6). Each dot represents a differentially expressed gene (DEG), colored by the corresponding microbial genus. Pie charts show the taxonomic composition of DEGs in each quadrant, and bar plots display the enriched KEGG pathways for genes correlated with transcriptional and microbial DNA methylation changes. (G) Classification and quantification of methyltransferases (MTases) and restriction enzymes, encompassing type I-IV, IIG restriction-modification (R-M) systems and orphan MTases. (H) Representative circular genome map of *Pseudoscardovia radai* (2.2 Mbp).

**FIGURE S6. The co-localization of differentially expressed genes and microbial DNA methylation in the rumen microbiome with divergent methane emissions.** (A-D) Genomic co-localization analysis of microbial DNA methylation sites and differentially expressed genes (DEGs) in *Prevotella lacticifex* (A), *Prevotella sp900316565* (B), *Ruminococcoides sp902773155* (C) and *Desulfovibrio sp900319575* (D).

**REFERENCES**

1. Hristov, Alexander N., Joonpyo Oh, Fabio Giallongo, Tyler Frederick, Holley Weeks, Patrick R. Zimmerman, Michael T. Harper, Rada A. Hristova, R. Scott Zimmerman, Antonio F. Branco. 2015. “The use of an automated system (GreenFeed) to monitor enteric methane and carbon dioxide emissions from ruminant animals.” *Journal of Visualized Experiments 7: 52904.* <https://doi.org/10.3791/52904>

2. Kong, Fanlin, Shuo Wang, Yijia Zhang, Chen Li, Dongwen Dai, Cheng Guo, Yajing Wang, et al. 2025. “Rumen microbiome associates with postpartum ketosis development in dairy cows: a prospective nested case-control study.” *Microbiome* 13: 69. <https://doi.org/10.1186/s40168-025-02072-3>

3. Gao, Mingkun, Youying Chen, Hao Fan, Shu Chen, Hui Wang, Wei Nie, Yuming Guo, Zengpeng Lv. 2024. “Transgenerational effects on the gene transcriptome of chicken liver.” *Animal Advances* 1: e003. https://doi.org/[10.48130/animadv-0024-0003](https://doi.org/10.48130/animadv-0024-0003)

4. Bolger, Anthony M, Marc Lohse, Bjoern Usadel. 2014. “Trimmomatic: a flexible trimmer for Illumina sequence data.” *Bioinformatics* 30: 2114-2120. https://doi.org/10.1093/bioinformatics/btu170

5. Chen, Shifu. 2023. “Ultrafast one‐pass FASTQ data preprocessing, quality control, and deduplication using fastp.” *iMeta* 2: e107. https://doi.org/10.1002/imt2.107

6. Li, Dinghua, Chi-Man Liu, Ruibang Luo, Kunihiko Sadakane, Tak-Wah Lam. 2015. “MEGAHIT: an ultra-fast single-node solution for large and complex metagenomics assembly via succinct de Bruijn graph.” *Bioinformatics* 31: 1674-1676. https://doi.org/10.1093/bioinformatics/btv033

7. Kang, Dongwan D, Jeff Froula, Rob Egan, Zhong Wang. 2015. “MetaBAT, an efficient tool for accurately reconstructing single genomes from complex microbial communities.” *PeerJ* 3: e1165. https://doi.org/10.7717/peerj.1165

8. Olm, Matthew R, Christopher T Brown, Brandon Brooks, Jillian F Banfield. 2017. “dRep: a tool for fast and accurate genomic comparisons that enables improved genome recovery from metagenomes through de-replication.” *The ISME Journal* 11: 2864-2868. https://doi.org/10.1038/ismej.2017.126

9. Hyatt, Doug, Gwo-Liang Chen, Philip F LoCascio, Miriam L Land, Frank W Larimer, Loren J Hauser. 2010. “Prodigal: prokaryotic gene recognition and translation initiation site identification.” *BMC Bioinformatics* 11: 119. https://doi.org/10.1186/1471-2105-11-119

10. Zheng, Jinfang, Qiwei Ge, Yuchen Yan, Xinpeng Zhang, Le Huang, Yanbin Yin. 2023. “dbCAN3: automated carbohydrate-active enzyme and substrate annotation.” *Nucleic Acids Research* 51: W115-W121. <https://doi.org/10.1093/nar/gkad328>

11. Buchfink, Benjamin, Chao Xie, Daniel H Huson. 2015. “Fast and sensitive protein alignment using DIAMOND.” *Nature Methods* 12: 59-60. https://doi.org/10.1038/nmeth.3176

12. Søndergaard, Dan, Christian N. S. Pedersen, Chris Greening. 2016. “HydDB: A web tool for hydrogenase classification and analysis.” *Scientific Reports* 6: 34212. <https://doi.org/10.1038/srep34212>

13. Schloss, Patrick D, Sarah L Westcott, Thomas Ryabin, Justine R Hall, Martin Hartmann, Emily B Hollister, Ryan A Lesniewski, Brian B Oakley, Donovan H Parks, Courtney J Robinson. 2009. “Introducing mothur: open-source, platform-independent, community-supported software for describing and comparing microbial communities.” *Applied and Environmental Microbiology* 75: 7537-7541. https://doi.org/10.1128/AEM.01541-09

14. Anderson, Marti J, Daniel CI Walsh. 2013. “PERMANOVA, ANOSIM, and the Mantel test in the face of heterogeneous dispersions: what null hypothesis are you testing?” *Ecological Monographs* 83: 557-574. https://doi.org/10.1890/12-2010.1

15. Wagner, Günter P., Koryu Kin, Vincent J. Lynch. 2012. “Measurement of mRNA abundance using RNA-seq data: RPKM measure is inconsistent among samples.” *Theory In Biosciences = Theorie In Den Biowissenschaften* 131: 281-285. <https://doi.org/10.1007/s12064-012-0162-3>

16. Robinson, Mark D., Davis J. McCarthy, Gordon K. Smyth. 2009. “edgeR: a Bioconductor package for differential expression analysis of digital gene expression data.” *Bioinformatics (Oxford, England)* 26: 139-140. <https://doi.org/10.1093/bioinformatics/btp616>

17. Li, Heng. 2018. “Minimap2: pairwise alignment for nucleotide sequences.” *Bioinformatics* 34: 3094-3100. <https://doi.org/10.1093/bioinformatics/bty191>

18. Kanaan, Georges, Jody W. Deming. 2025. “Multiple roles of DNA methylation in sea-ice bacterial communities and associated viruses.” *The ISME Journal* 19: wraf198. https://doi.org/10.1093/ismejo/wraf198

19. Crits-Christoph, Alexander, Shinyoung Clair Kang, Henry H Lee, Nili Ostrov. 2023. “MicrobeMod: A computational toolkit for identifying prokaryotic methylation and restriction-modification with nanopore sequencing.” *BioRxiv*. https://doi.org/10.1101/2023.11.13.566931
